# Observation and imitation of object-directed hand movements in Parkinson’s disease

**DOI:** 10.1038/s41598-023-42705-x

**Published:** 2023-10-31

**Authors:** Judith Bek, Emma Gowen, Stefan Vogt, Trevor J. Crawford, Ellen Poliakoff

**Affiliations:** 1 School of Psychology, University College Dublin, Dublin, Ireland; 2 Centre for Motor Control, Faculty of Kinesiology and Physical Education, University of Toronto, Toronto, Canada; 3 Division of Psychology Communication and Human Neuroscience, School of Health Sciences, University of Manchester, Manchester, UK; 4 Department of Psychology, Lancaster University, Lancaster, UK

**Keywords:** Human behaviour, Parkinson's disease

## Abstract

Action observation and imitation may facilitate movement in Parkinson’s disease (PD). People with PD have been found to imitate intransitive actions similarly to neurologically healthy older adults, but their imitation of object-directed hand movements has not previously been investigated using kinematic measures. The present study examined observation and imitation of object-directed hand movements in 18 participants with PD and 21 neurologically healthy age-matched control participants. Participants observed and immediately imitated sequences showing a human hand reaching for and transferring an object between horizontal positions. Both groups significantly modulated their finger movements, showing higher vertical amplitude when imitating elevated compared to direct trajectories. In addition, movements were lower in vertical amplitude and higher in velocity when imitating the reaching segment than the transfer segment. Eye-tracking revealed that controls made smaller saccades when observing predictable than unpredictable elevated movements, but no effects of predictability on eye movements were found for the PD group. This study provides quantitative evidence that people with mild to moderate PD can imitate object-directed hand movement kinematics, although their prediction of such movements may be reduced. These findings suggest that interventions targeting object-directed actions may capitalize on the ability of people with PD to imitate kinematic parameters of a demonstrated movement.

## Introduction

Parkinson’s disease (PD) is a neurodegenerative disorder that affects an estimated 10 million people worldwide, and is rapidly increasing in prevalence^1^. The neuropathology of PD involves depletion of dopamine in the basal ganglia, resulting in multiple motor impairments including difficulties with gait, balance, posture, functional mobility, and dexterity. Activities of daily living, such as eating and dressing, as well as other everyday tasks that require fine motor control, are impacted in PD^2,3^, and dexterity has been highlighted as a priority area for research by those living with the condition^4^.

Observation of human movement has been explored as a therapeutic approach for neurological conditions including PD and stroke^5–8^, based on evidence that action observation (AO) enhances performance and learning in healthy populations^9,10^. Overlapping neural networks are found to be activated by observation and execution of actions^11,12^, and imitation—which involves both observation and physical execution of an action—recruits a wider network of brain regions^11^. AO combined with physical practice (imitation) therefore offers a promising technique to promote activation of the motor system and to support the maintenance of functional ability in PD^6^. Recent studies have demonstrated that AO and imitation are relatively preserved among individuals with PD^13–15^. In particular, people with PD imitated the timing and distance of intransitive (non-object-directed) pointing movements in a similar manner to neurologically healthy age-matched participants^14^ and imitated the trajectory of a human hand movement more closely than that of a non-biological object^15^, although the extent to which people with PD modulate the trajectory of imitated hand movements may be somewhat reduced^15^. Additionally, improvements in motor symptoms such as gait and balance have been reported following AO interventions in people with PD^16–18^. Preliminary evidence from pilot studies has also indicated potential improvements in functional independence^18,19^ and functional hand movements^20^ following AO-based training with object-directed actions in people with PD. However, mechanisms of observation and imitation of object-directed actions have not been directly assessed in people with PD.

In neurologically healthy participants, observation and execution of object-directed actions, such as reaching and grasping, activate areas of the posterior parietal cortex more strongly than intransitive hand gestures^21,22^. The basal ganglia also have an important role in the AO network^23^, and appear to be particularly involved in the observation and execution of object manipulation actions such as reaching, grasping, and relocating^24^, suggesting that basal ganglia pathology in PD may lead to difficulties in imitating object-directed actions.

Neurologically healthy adults have been found to imitate intransitive actions more accurately than object-directed actions^25^. According to goal-directed accounts of imitation, observed actions are represented based on a hierarchy of goals, such that target objects or endpoints may be prioritized over the kinematics of the movement^26,27^. Consistent with this theory, neurologically healthy participants show reduced imitation of kinematics in the presence of visible movement endpoints^28,29^. Given the importance of object-directed actions for everyday activities, and the potential impact of basal ganglia pathology on such actions, it is important to understand how AO and imitation of object-directed actions may be affected by PD.

There is some evidence to suggest that the processes involved in observation and imitation of object-directed actions may be altered in PD. For example, behavioural studies have reported reduced accuracy when people with PD imitated pantomimed transitive actions^30,31^. Moreover, a neurophysiological study found that when individuals with PD were asked to observe, imagine, or imitate a cutting action while holding a pair of scissors, motor evoked potentials of the hand muscles were facilitated only during the imitation task, whereas an age-matched neurologically healthy control group exhibited corticomotor facilitation across all three tasks compared to a rest condition^32^. However, kinematic measures of imitation of object-directed actions have not been studied.

The present study used motion tracking to investigate imitation of movement trajectories in the context of object-directed hand movements in people with PD compared to a neurologically healthy age-matched control group. Similar to previous studies of people with PD^14,15^ and without PD^29,33^, an exaggerated elevated trajectory (i.e., higher than necessary to reach the target endpoint) was compared with a more direct trajectory between target positions, to ensure that participants would attend to the kinematics of the movement rather than just the endpoints (see Ref.^34^).

Based on previous findings from studies of AO and imitation of intransitive actions in PD^14,15^, it was hypothesised that participants with PD would imitate the trajectory of observed movements by modulating the vertical amplitude of their own hand movements in response to stimuli depicting trajectories of different heights, although they might exhibit reduced modulation relative to age-matched control participants^15^. Alternatively, if the basal ganglia have a particular role in observing and executing object-directed actions^24^, people with PD may have greater difficulty in imitating such actions.

To further examine mechanisms of object-directed imitation, the movement sequences to be imitated included two segments, in which the model first reached towards an object and picked it up, then transferred the object to a new location. The “reach” segment thus involved a movement towards a visible target, which was expected to result in reduced imitation of the kinematics for both groups relative to the “transfer” segment, in which the kinematics may be prioritized and attended to more closely in the absence of a visible target object^26,27^. Additionally, it was speculated that imitated reach movements might be faster and smoother than imitated transfer movements for both groups, anticipating that the visible target object would facilitate a more direct movement towards the perceived or remembered location of the object^27,28^. Although participants observed the model’s hand grasping and picking up the object, they did not physically manipulate an object in their own movement space. This was to ensure that the movements executed by the participant were based on a representation of the observed action (i.e., imitation), rather than simply being driven by reaching for the object, which could provide a direct affordance or visual cue. Nonetheless, if people with PD have a particular difficulty with object-directed actions, they may still rely more on the object as a cue during observation and attend less than controls to the kinematics of the movement, subsequently exhibiting a greater difference in imitation between reach and transfer segments, compared to the control group.

Finally, eye movements during action observation were recorded to explore potential differences between groups in action observation and prediction. It was hypothesised that fewer and smaller eye movements might be made when observing predictable compared to unpredictable actions, based on previous findings that both individuals with PD and neurologically healthy older adults made fewer and smaller eye movements when watching a moving finger than a moving shape, which might reflect greater ongoing prediction of the movement^15^. It was also anticipated that predictability effects might be greater for elevated than direct trials, since participants may attend more closely to the kinematics of the atypical elevated trajectory. If processes of AO and imitation of object-directed actions are altered in people with PD, they might exhibit differences in eye movements, such as reduced effects of predictability, compared to age-matched control participants.

## Methods

### Participants

Eighteen individuals diagnosed with idiopathic PD (5 female and 13 male participants; mean age 63.7 years, *SD* = 6.8) were recruited through Parkinson’s UK and local neurology clinics. The mean time since diagnosis was 7.7 years (*SD* = 4.6) and participants had mild to moderate symptoms based on the Hoehn and Yahr scale^2^ (*M* = 2, *SD* = 0.5), with a mean Unified Parkinson’s Disease Rating Scale (UPDRS-MDS^3^) motor score of 42.8 (*SD* = 12.8). Participants with PD remained on their regular dopaminergic medication during testing and none had a history of surgical intervention. The control group consisted of 21 older adults with no history of neurological injury or illness (10 female and 11 male participants; mean age 67.3 years, *SD* = 7.3) who were recruited through a volunteer list and local community groups. All participants except two in the PD group were right-handed. There was no significant difference in age between the groups (t(37) = 1.76; p = 0.087), and age was not found to contribute significantly to imitation effects, so was not included in further analysis.

The study was approved by a UK National Health Service Research Ethics Committee. All procedures were conducted in accordance with the requirements of the ethical approval and the Declaration of Helsinki. Written informed consent was obtained from all participants.

### Stimuli and procedure

**Figure 1:**
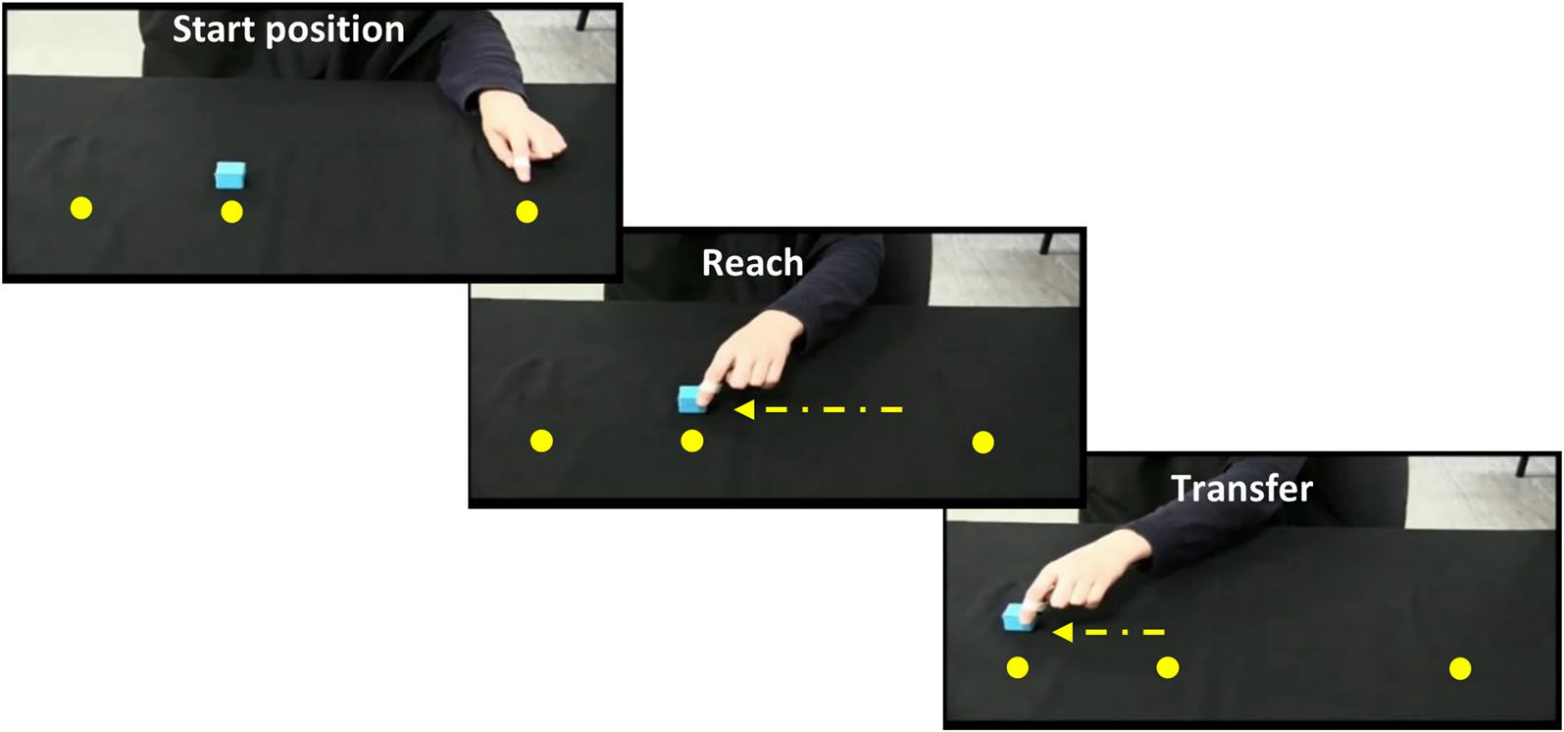
Stimulus videos depicted a human hand reaching for and moving a small cube between 3 of 4 possible positions spaced 150 mm apart (example shows sequence 4-2-1), following either a direct or elevated trajectory. Participants observed and then immediately imitated the sequence but without physically manipulating an object (the object was not present in their own movement space). Note that the circles and arrows indicating the sequence are shown for illustration only and no target markers were visible during the task. Example stimulus videos are available at https://osf.io/ysbrj/.

Participants observed video recordings of simple movement sequences depicted by a human hand and then immediately imitated the movements using their dominant hand. The video was shown as a mirror image, such that right-handed participants viewed a left-handed stimulus and left-handed participants viewed a right-handed stimulus. In each sequence, the hand reached for and grasped a small cube-shaped object and then transferred it to another location (see Fig. 1).

The sequences involved movements between three of four possible positions (e.g., starting at position 4, reaching for an object at position 2 and transferring the object to position 1) at intervals of 150 mm along a horizontal movement space. Each sequence consisted of one longer movement (300 mm; e.g., positions 4–2) and one shorter movement (150 mm; e.g., positions 2–1). To minimise variability and noise in the data, only the longer segment from each sequence was included in the analysis: this was the reach segment in 50% of trials (sequences 3-1-2; 4-2-1) and the transfer segment in 50% of trials (sequences 3-4-2; 4-3-1). Within each trial, both parts of the sequence followed either a direct trajectory, with a vertical amplitude of approximately 85 mm at the apex of the movement, or an elevated trajectory, with a vertical amplitude of approximately 195 mm. Video clips were approx. 3 s in duration and were followed immediately by a “beep” sound signaling for the participant to commence their movement.

Stimuli were projected at life-size onto a 530 mm × 300 mm screen, positioned approximately 700 mm from the participant. As noted above, to avoid the potential use of the object as a direct visual cue, participants did not physically manipulate an object in their own movement space, but were instructed to perform the movement as if the object was present: “Watch the video carefully, and then after the beep, copy what you have seen as closely as you can in terms of the timing and size of the movement. Please perform the action as if you were actually moving the block”. A short practice block of four trials was followed by 60 test trials, presented in two blocks of 30. Each block contained 10 elevated trajectory trials and 10 direct trajectory trials (20 of each type in total). The remaining 10 trials in each block depicted slightly faster direct movements to examine potential modulation of timing, but the difference in peak velocity was very subtle (108 mm/s) and preliminary analysis revealed no significant differences in imitation of movement duration or peak velocity between these faster trials and the direct trials in either group, so the faster trials were omitted from further analysis. The order of trials within each block was randomized and a short break was provided halfway through each block.

A motion sensor was attached to the intermediate phalanx of the index finger of the participant’s dominant hand. Hand position was tracked in X, Y, and Z axes using a Polhemus Fastrak^®^ electromagnetic motion capture system at a sampling rate of 120 Hz. Eye movements were recorded while participants observed the hand movement sequences, using an Eyelink 1000 Plus eye tracker (SR Research Ltd.) with remote monocular pupil capture at a sampling rate of 500 Hz, with a spatial resolution of 0.1° and saccade detection threshold of 30°/s. A nine-point calibration was performed with each participant prior to the experiment.

### Data processing and statistical analysis

Kinematic data from trials in which the movement sequence was correctly imitated (i.e., the positions were moved to in the correct order) were extracted and analysed using MATLAB version 7.10.0. The kinematic measures included in the analysis were: vertical amplitude, peak velocity, and dimensionless jerk (a measure of movement smoothness^35^). Missing data (incomplete or missing trials) and errors (incorrect sequences) were removed, constituting 7% of trials in the PD group and 1% in the control group. Outliers were then identified and removed using the standard deviation procedure described by van Selst and Jolicouer^36^. This resulted in the exclusion of 1.81% of datapoints from the PD group and 2.33% from the control group. The kinematics of the longer movement in each trial were then analysed using linear mixed-effects modelling (LMM). The factors Group (PD, control), Trajectory (elevated, direct), and Segment (reach, transfer) were included as fixed effects with random intercept effects for Participants. To allow for greater estimation of variance components, random slopes for Trajectory or Segment were also included where these improved the fit of the model (i.e., Trajectory for vertical amplitude; Segment for horizontal amplitude, peak velocity, and dimensionless jerk). Models were fitted using the maximum likelihood procedure with the Satterthwaite adjustment method. Significant interactions were further analysed using t-tests.

Eye movements during observation of the movement sequences were analysed for 16 participants in the PD group and 20 in the control group (recordings were incomplete or unusable for 2 PD participants and one control group participant; see Ref.^37^ for discussion of challenges of eye tracking with these populations). Fixations and saccades were analysed to identify effects of the predictability of the observed transfer movements. While the direction of the “reach” segment was always predictable (because the model reached towards a visible object), the “transfer” segment was considered predictable if this segment started from the furthest endpoint; i.e., position 4 (where the hand could only move in one direction), or unpredictable if it started from position 3 (where either a leftward or rightward movement was possible). Equal numbers of predictable and unpredictable transfer movements were included across trials.

Trials where loss of capture (e.g., due to excessive blinking) exceeded 30% were removed from the eye movement data (7.81% of trials in the PD group; 3.5% in the control group). Removal of outliers then resulted in the exclusion of a further 3.36% of datapoints from the PD group and 3.69% from the control group.

Fixations and saccades were analysed using LMM, with fixed factors of Group, Trajectory, and Predictability, random intercepts for Participants, and random slopes for Predictability. Statistical analyses were conducted in R^38^ using the package lme4^39^.

Examples of kinematic and eye movement time series data for complete trials are provided at https://osf.io/ysbrj/.

## Results

The best-fitting models for each dependent variable in the kinematic and eye movement analyses are summarised below. Full details of model structures, parameters, and effects are provided in supplementary materials.

### Kinematic analysis

**Figure 2:**
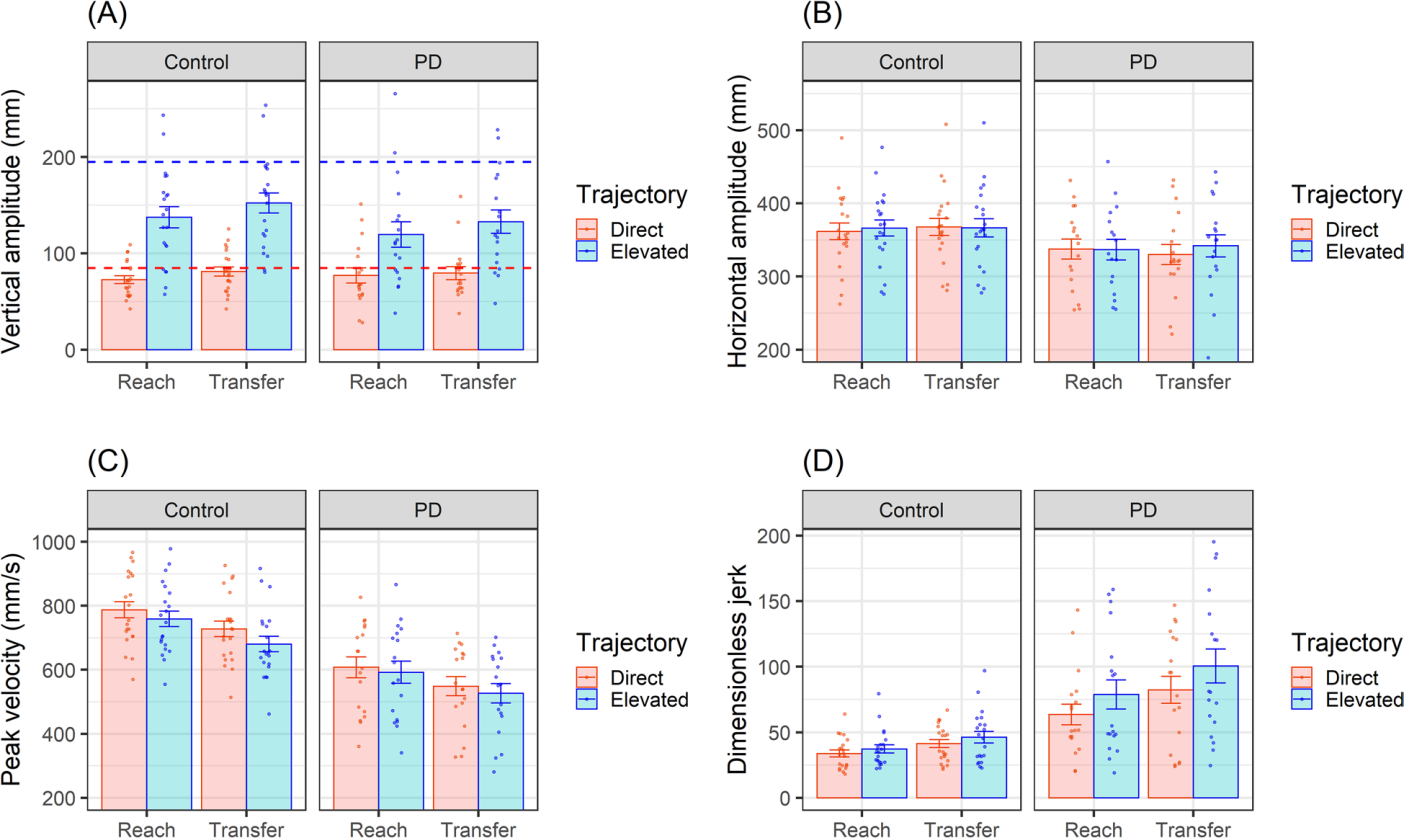
Kinematic measures during imitation of object-directed actions: each measure is presented for imitation of elevated vs. direct trajectories in reach and transfer segments of the sequences. Plots show means with SEM error bars; dots represent individual participants. (**A**) Vertical amplitude was significantly higher for elevated vs. direct trials (indicating imitation of trajectory) and for transfer vs. reach segments. There was a non-significant trend for reduced vertical amplitude modulation in the PD group. Reference lines indicate model kinematics for the direct (red dashed line) and elevated (blue dashed line) trajectories. (**B**) Horizontal amplitude did not differ significantly between groups, but movements were longer in elevated vs. direct trials in the transfer segment in the PD group. (**C**) Peak velocity was significantly higher in the control group, as well as for direct vs. elevated trials and reach vs. transfer segments. (**D**) Dimensionless jerk was significantly higher in the PD group, particularly for elevated movements.

Analysis of vertical amplitude (Fig. 2A) revealed a significant effect of Trajectory (b = 64.0, SE = 8.69, t(44.73) = 7.36; p < 0.001), such that amplitude was greater when imitating elevated movements (M = 137 mm, SD = 60.9 mm) than direct movements (M = 78 mm, SD = 33.6 mm), indicating that participants modulated the trajectory of their own hand movements in response to differences in the observed movement trajectory. There was also a significant effect of Segment (b = 8.24, SE = 3.13, t(1401.91) = 2.63; p = 0.0086), reflecting higher amplitude movements in the transfer segment (M = 112 mm, SD = 56.8 mm) than the reach segment (M = 103 mm, SD = 57.3 mm). There was no significant effect of Group (b = 3.83, SE = 7.48, t(47.76) = 0.51, p = 0.61), but the interaction between Group and Trajectory showed a non-significant trend (b = -21.39, SE = 12.81, t(44.95) = -1.67; p = 0.1), reflecting a slight reduction of modulation in the PD group (M = 47.98 mm, SD = 38.78 mm) compared to the control group (M = 67.96 mm, SD = 40.02 mm).

For horizontal amplitude (Fig. 2B), there were no significant main effects of Trajectory (b = 4.81, SE = 3.82, t(1398.30) = 1.26, p = 0.21), Segment (b = 6.04, SE = 5.37, t(65.46) = 1.13, p = 0.26), or Group (b = -26.46, SE = 16.94, t(41.20) = -1.56, p = 0.13), but there was a significant interaction between Group, Trajectory and Segment (b = 18.33, SE = 7.97, t(1397.45) = 2.30, p = 0.02). In the PD group, movements were significantly longer in elevated than direct trials in the transfer segment (elevated M = 341 mm, SD = 77.4, direct M = 330 mm, SD = 72.4 mm; t(17) = − 2.38; p = 0.029) but not the reach segment (elevated M = 335 mm, SD = 68.2 mm, direct M = 336 mm, SD = 64.7 mm; t(16) = 0.25; p = 0.81).

Analysis of peak velocity (Fig. 2C) showed significant main effects of Trajectory (b = − 27.29, SE = 10.77, t(114.88) = − 2.54; p < 0.001), Segment (b = − 60.60, SE = 9.65, t(1413.33) = − 6.28; p < 0.001) and Group (b = − 180.57, SE = 36.93, t(42.19) = − 4.89; p < 0.001). Overall peak velocity was higher in the control group (M = 740 mm/s, SD = 150 mm/s) than the PD group (M = 566 mm/s, SD = 158 mm/s). Peak velocity was higher when imitating direct movements (M = 676 mm/s, SD = 180 mm/s) than elevated movements (M = 646 mm/s, SD = 172 mm/s), and for reach segments (M = 695 mm/s, SD = 177 mm/s) compared to transfer segments (M = 628 mm/s, SD = 170 mm/s).

For dimensionless jerk (Fig. 2D), there was no significant main effect of Trajectory (b = 3.55, SE = 3.55, t(1383.05) = 1.0, p = 0.32) or Segment (b = 7.44, SE = 5.37, t(60.11) = 1.39, p = 0.17), but there was a significant main effect of Group (b = 29.6, SE = 9.16, t(46.15) = 3.23; p = 0.0023), reflecting higher overall jerk (i.e., less smooth movements) in the PD group (M = 83.0, SD = 64.4) than the control group (M = 39.9, SD = 23.7). There was also a significant interaction between Group and Trajectory (b = 12.24, SE = 5.24, t(1382.46) = 2.34; p = 0.02): as illustrated in Fig. 2D, while movements were smoother overall for direct trials than elevated trials, the difference in jerk between direct and elevated trials was greater in the PD group (elevated M = 91.9, SD = 73.1; direct M = 74.2, SD = 53.2) than the control group (elevated M = 42.2, SD = 26.7; direct M = 37.8, SD = 20.1); t(47.5) = − 2.79; p = 0.0075; d = 0.65.

All other main effects and interactions for the kinematic measures were non-significant (all p > 0.1; see Supplementary materials Table 1).

### Eye movements

**Figure 3:**
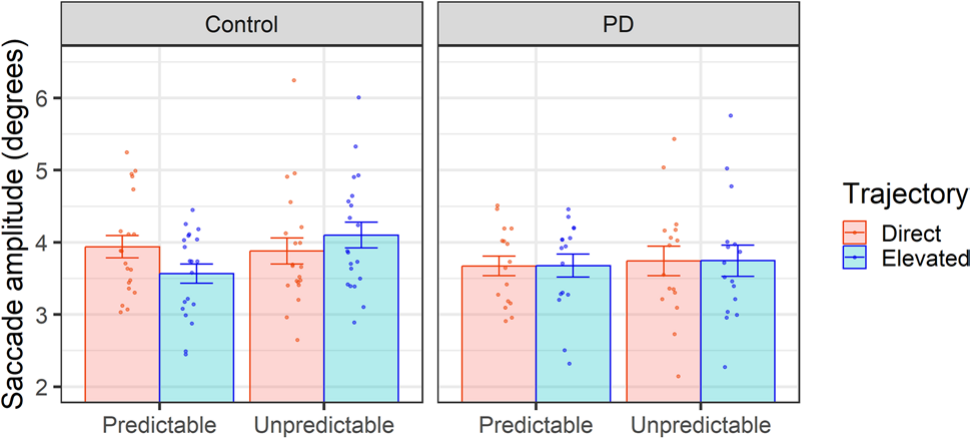
Saccade amplitude during observation of object-directed actions was significantly reduced for predictable vs. unpredictable transfer movements in the control group, specifically in trials with an elevated trajectory. Plots show means with SEM error bars; dots represent individual participants.

Analysis of saccade amplitude (Fig. 3) showed significant main effects of Trajectory (b = 0.037, SE = 0.10, t(1302.05) = 3.70, p < 0.001) and Predictability (b = 0.53, SE = 0.16, t(56.10) = 3.37, p = 0.0014), but no significant main effect of Group (b = 0.11, SE = 0.21, t(47.36) = 0.55, p = 0.58). There were significant interactions between Group and Trajectory (b = − 0.38, SE = 0.15, t(1300.23) = − 2.53, p = 0.012), Trajectory and Predictability (b = − 0.58, SE = 0.14, t(1299.9) = − 4.13, < 0.001), and a 3-way interaction between Group, Trajectory, and Predictability (b = 0.59, SE = 0.21, t(1299.4) = 2.79, p = 0.0054). T-tests indicated that participants in the control group exhibited significantly smaller saccades when observing predictable compared to unpredictable movements in elevated trials (predictable M = 3.57, SD = 1.02; unpredictable M = 4.08, SD = 1.24; t(19) = − 3.62; p = 0.0018; d = 0.81) but not in trials with a direct trajectory (predictable M = 3.92, SD = 1.22; unpredictable M = 3.88, SD = 1.20; t(19) = 0.31; p = 0.76; d = 0.07). The PD group showed no significant effect of predictability for either the elevated trials (predictable M = 3.66, SD = 1.06; unpredictable M = 3.74, SD = 1.23; t(15) = − 0.365; p = 0.72; d = 0.09) or direct trials (predictable M = 3.65, SD = 1.04; unpredictable M = 3.72, SD = 1.28; t(15) = − 0.43; p = 0.68; d = 0.11).

For all other eye movement measures (saccade count, fixation count, and fixation duration) there were no significant main effects or interactions (all p > 0.09; see supplementary materials, Table 2).

## Discussion

The present study demonstrated that individuals with mild to moderate PD were able to imitate object-directed actions by modulating the trajectory of their hand movements according to differences in the observed trajectory. People with PD showed a similar pattern of imitation to neurologically healthy age-matched control participants when imitating both reach and transfer segments of the hand movement sequences. These results extend previous findings indicating the ability of people with mild to moderate PD to imitate intransitive hand movements^14,15^, providing quantitative evidence that their imitation of object-directed movements is also relatively preserved. Although modulation of kinematics was not significantly reduced in people with PD compared to the control group, there was a non-significant trend towards reduced modulation in the PD group. Previous studies of intransitive hand movements have not consistently found a significant difference in imitation between PD and control groups^14,15^. It is therefore possible that a subtle deficit in imitation exists, which the present and previous studies were not sufficiently powered to detect.

It should also be noted that the overall extent of vertical amplitude did not differ significantly between groups, although peak velocity was lower and jerk was higher in the PD group, likely reflecting effects of PD symptoms such as bradykinesia, tremor, and rigidity. The fact that vertical amplitude did not differ overall between groups suggests that action observation may be particularly effective in maintaining movement size in people with PD, although this is speculative without a comparison condition in which movements were performed without action observation.

In addition, horizontal amplitude (distance of movement) did not differ significantly overall between groups, but the PD group exhibited longer transfer movements in elevated compared to direct trials. This may reflect the higher vertical amplitude of participants' imitated elevated movements in the transfer segment than the reach segment, which appears to correspond to an increase in horizontal amplitude for the PD group. This finding suggests that increasing the vertical amplitude of movements may indirectly also promote maintenance of horizontal amplitude.

Despite the similar modulation of kinematics between groups, a difference was found in eye movements when observing object-directed hand movements. Specifically, neurologically healthy participants showed an effect of predictability when observing movements with an elevated trajectory (smaller saccades for predictable vs. unpredictable movements), but the PD group did not exhibit any effects of predictability on their eye movements, suggesting that action prediction may be reduced in PD. This may relate to alterations in the perception of biological motion, as indicated by findings showing impaired perception of body movements from point-light displays in both medicated and unmedicated participants with PD^40,41^. It is also possible that reduced prediction is caused by difficulties with sequence learning in PD^42^. However, the present findings contrast with previous research that found no differences between groups in eye movements when observing intransitive hand movements^15^. Further research is needed, with more fine-grained analysis of oculomotor measures (e.g., acceleration and corrective saccades) and additional manipulations of predictability, to understand whether eye movements during action observation reflect action prediction mechanisms in people with PD and neurologically healthy older adults.

To further understand potential effects of PD on imitation of object-directed actions, the present study examined kinematics when imitating different segments of the action that involved reaching for the object and transferring it to a new location. Participants in both groups made faster, lower amplitude movements when imitating the reaching segment than the transfer segment. This may be explained in relation to goal-directed mechanisms in imitation, whereby the target object provides a higher-level goal than the kinematics of the movement itself^26,27^, resulting in faster and more direct movements during imitation. It is noteworthy that this difference between reaching and transferring segments was found even though the object was not physically present during action execution, suggesting that the two segments were encoded differently during observation, or that participants imagined (i.e., mentally simulated) reaching for the object in their own movement space. However, the absence of an interaction between trajectory and segment indicates that *modulation* of the kinematics was not reduced when imitating movements towards a visible endpoint (reach segment) compared to transfer movements without a visible endpoint, as might be expected based on previous findings^28,29^. A greater difference in kinematic imitation may therefore be found if participants reached for a real object.

There is considerable evidence that external visual cues can be effective in facilitating movement in people with PD, although this literature is largely focused on cueing of gait rather than upper limb movements^43,44^. Together with previous findings, the present results indicate that while visual cues (such as objects to reach towards) could increase the velocity and smoothness of hand movements, observation and imitation of human kinematics (e.g., the trajectory of an action) may instead influence other aspects of movement such as amplitude^14,15^. It is possible that a more complex pattern would emerge when using objects associated with specific actions (affordances). Indeed, previous work has indicated that people with PD are as responsive, or more so, than people without PD to observing objects associated with grasping actions such as handles (i.e., they show effects of affordances; for a review see Ref.^45^).

The results of this study demonstrate that people with PD are able to imitate the trajectory of reach and transfer movements in a similar manner to neurologically healthy individuals, even if the extent of imitation may be somewhat reduced. This indicates the potential benefit of AO-based interventions for people with PD, which could help to preserve or improve the performance of object-directed actions. This is also indicated by preliminary evidence from intervention studies showing that training with AO, particularly when combined with motor imagery and physical execution, may enhance the performance of daily activities in individuals with PD, including manual actions using everyday objects^18,20^ which may capitalize on responses to affordances^45^. The efficacy of combined AO and motor imagery has also been demonstrated in other populations and at different levels of skill acquisition^46^.

In conclusion, the present study demonstrated that individuals with mild to moderate PD were able to modulate the amplitude of their hand movements by imitating the kinematics of object-directed actions, exhibiting a similar pattern to neurologically healthy age-matched participants. Future studies should examine observation and imitation of more complex object-directed actions (including actions involving multiple objects) and determine the effectiveness of AO-based training to augment everyday object-directed activities that are central to functional independence for people with PD.

### Supplementary Information

Supplementary Tables.

## Data Availability

Anonymised data will be made available on reasonable request from the corresponding author.
