# Supplementary material for: Observation and imitation of object-directed hand movements in Parkinson’s disease
Source: Sci Rep. 2023 Oct 31;13:18749. doi: 10.1038/s41598-023-42705-x (PMC10618260; doi:10.1038/s41598-023-42705-x)
Supplement: Supplementary file 1 — Supplementary Tables. [file 41598_2023_42705_MOESM1_ESM.pdf]

Table 1. Model summaries for the best-fitting linear mixed-effects models for each kinematic measure (vertical amplitude, horizontal amplitude, peak velocity, and dimensionless jerk).

Model	Predictors (b, SE, df, t, p)	Model df	BIC	AIC	LogLik	Deviance	Marginal/ Conditional R^2
**Vertical amplitude: Group* Trajectory*Segment + (1 + Trajectory | Participant)**		1467	14690.8	14627.2	-7301.6	14603.2	.29/.70
**(Intercept)**	**73.12, 5.07, 47.29, 14.42, <.001**						
Group: PD	3.83, 7.48, 47.76, .51, .61						
**Trajectory: Elevated**	**64.0, 8.69, 44.73, 7.36, <.001**						
**Segment: Transfer**	**8.24, 3.13, 1401.91, 2.63, .0086**						
Group* Trajectory	-21.39, 12.81, 44.95, -1.67, .10						
Group* Segment	-5.45, 4.64, 1402.39, -1.17, .24						
Trajectory* Segment	6.84, 4.41, 1401.4, 1.55, .12						
Group* Trajectory* Segment	3.86, 6.56, 1401.95, .59, .56						
**Horizontal amplitude: Group* Trajectory*Segment + (1 + Segment | Participant)**		1461	15193.1	15129.6	-7552.8	15105.6	.053/.68
**(Intercept)**	**361.62, 11.50, 41.03, 31.45, <.001**						
Group: PD	-26.46, 16.94, 41.20, -1.56, .13						
Trajectory: Elevated	4.81, 3.82, 1398.30, 1.26, .21						

Model	Predictors (b, SE, df, t, p)	Model df	BIC	AIC	LogLik	Deviance	Marginal/ Conditional R^2
Segment: Transfer	6.04, 5.37, 65.46, 1.13, .26						
Group* Trajectory	-5.99, 5.68, 1397.54, -1.05, .29						
Group* Segment	-10.81, 7.96, 67.26 -1.36, .18						
Trajectory* Segment	-5.91, 5.36, 1397.30, -1.10, .27						
**Group* Trajectory* Segment**	**18.33, 7.97, 1397.45, 2.30, .02**						
**Peak velocity: Group* Trajectory*Segment + (1 + Segment | Participant)**		1478	18094.0	18030.4	-9003.2	18006.4	.28/.69
**(Intercept)**	**787.89, 25.06, 42.02, 31.44, <.001**						
**Group: PD**	**-180.57, 36.93, 42.19, -4.89, <.001**						
**Trajectory: Elevated**	**-27.29, 10.77, 114.88, -2.54, .013**						
**Segment: Transfer**	**-60.60, 9.65, 1413.33, -6.28, <.001**						
Group* Trajectory	10.81, 14.12, 1413.31, .77, .44						
Group* Segment	.73, 21.22, 63.33, .035, .97						
Trajectory* Segment	-19.86, 13.34, 1412.89, -1.49, .14						
Group* Trajectory* Segment	13.73, 19.82, 1413.54, .69, .49						

Model	Predictors (b, SE, df, t, p)	Model df	BIC	AIC	LogLik	Deviance	Marginal/ Conditional R^2
**Dimensionless jerk: Group* Trajectory*Segment + (1 + Segment | Participant)**		1448	14790.0	14726.5	-7351.3	14702.2	.20/.53
**(Intercept)**	**33.78, 6.21, 45.85, 5.44, <.001**						
**Group: PD**	**29.60, 9.16, 46.15, 3.23, .0023**						
Trajectory: Elevated	3.55, 3.55, 1383.05, 1.0, .32						
Segment: Transfer	7.44, 5.37, 60.11, 1.39, .17						
**Group* Trajectory**	**12.24, 5.24, 1382.46, 2.34, .020**						
Group* Segment	11.62, 7.94, 61.30, 1.46, .15						
Trajectory* Segment	1.64, 4.97, 1381.35, .33, .74						
Group* Trajectory* Segment	1.71, 7.38, 1382.68, .23, .82						

Note: Significant effects (p <.05) shown in bold.

Table 2. Model summaries for the best-fitting linear mixed-effects models for each eye movement measure (saccade count, saccade amplitude, fixation count, and fixation duration).

Model	Predictors (b, SE, df, t, p)	Model df	BIC	AIC	LogLik	Deviance	Marginal/ Conditional R^2
**Saccade count: Group* Predictability* Trajectory + (1 + Predictability | Participant)**		1354	7023.3	6945.0	-3457.5	6915.0	.012/.14
**(Intercept)**	**10.77, .73, 37.17, 14.83, <.001**						
Group: PD	.44, 1.09, 37.2, .41, .69						
Trajectory: Direct	-.046, .31, 293.99, -.15, .88						
Predictability: Unpredictable	.63, .37, 75.29, 1.68, .097						
Group* Trajectory	.64, .46, 295.87, 1.39, .17						
Group* Predictability	-.41, .56, 75.14, -.74, .46						
Predictability* Trajectory	-.12, .41, 1298.21, -.30, .76						
Group* Predictability* Trajectory	-.54, .62. 1298.16, -.88, .38						
**Saccade amplitude: Group* Predictability* Trajectory + (1 + Predictability | Participant)**		1358	4032.6	3969.9	-1973.0	3945.9	.012/.32
**(Intercept)**	**3.56, .14, 47.23, 25.84, <.001**						
Group: PD	.11, .21, 47.36, .55, .58						
**Trajectory: Direct**	**.037, .10, 1302.05, 3.70, <.001**						

Model	Predictors (b, SE, df, t, p)	Model df	BIC	AIC	LogLik	Deviance	Marginal/ Conditional R^2
**Predictability: Unpredictable**	**.53, .16, 56.10, 3.37, .0014**						
**Group* Trajectory**	**-.38, .15, 1300.23, -2.53, .012**						
Group* Predictability	-.46, .24, 55.94, -1.96, .055						
**Predictability* Trajectory**	**-.58, .14, 1299.9, -4.13, <.001**						
**Group* Predictability* Trajectory**	**.59, .21, 1299.4, 2.79, .0054**						
**Fixation count: Group* Predictability* Trajectory + (1 + Predictability | Participant)**		1363	7059.9	6997.2	-3488.3	6973.2	.005/.57
**(Intercept)**	**11.48, .74, 38.84, 15.46, <.001**						
Group: PD	.44, 1.11, 38.92, .40, .70						
Trajectory: Direct	.053, .29, 1307.07, .18, .86						
Predictability: Unpredictable	.60, .38, 73.34, 1.58, .12						
Group* Trajectory	.55, .44, 1305.39, 1.25, .21						
Group* Predictability	-.41, .57, 73.31, -.71, .48						
Predictability* Trajectory	1.13, .41, 1305.23, -.31, .76						
Group* Predictability* Trajectory	.42, .62. 1304.83, -.67, .50						

Model	Predictors (b, SE, df, t, p)	Model df	BIC	AIC	LogLik	Deviance	Marginal/ Conditional R^2
**Fixation duration: Group* Predictability* Trajectory + (1 + Predictability | Participant)**		1356	17372.4	17435.1	-8674.2	17348.4	.01/.55
**(Intercept)**	**396.62, 33.51, 39.1, 11.84, <.001**						
Group: PD	48.26, 50.25, 39.06, .96, .34						
Trajectory: Direct	6.49, 13.39, 1301.68, .49, .63						
Predictability: Unpredictable	-11.38, 15.1, 94.7, -.75, .45						
Group* Trajectory	-.24.60, 20.0, 1299.09, -1.23, .22						
Group* Predictability	-2.06, 22.65, 94.67, -.09, .93						
Predictability* Trajectory	8.58, 18.78, 1298.66, .46, .65						
Group* Predictability* Trajectory	1.44, 28.3. 1298.32, .051, .96						

Note: Significant effects (p <.05) are shown in bold.
